# Emergency Tracheal Resection and Anastomosis for Life-Threatening Intratracheal B-Cell Lymphoma in a Cat: A Case Report

**DOI:** 10.3390/vetsci13101032

**Published:** 2026-09-29

**Authors:** Ho-Jun Hwang, Tae-Hee Kim, Seong-Yong Park, Chang-Hwan Moon, Jung-Moon Kim, Hwi-Yool Kim

**Affiliations:** 1 Department of Veterinary Surgery, College of Veterinary Medicine, Konkuk University, Seoul 05029, Republic of Korea; hwanghj88@gmail.com (H.-J.H.); kimjungm418@gmail.com (J.-M.K.); 2 Prime Animal Medical Center, Seoul 05251, Republic of Korea; 3 Gyeyang Sky Animal Medical Center, Incheon 21079, Republic of Korea; bighee92@naver.com (T.-H.K.); kainblus@naver.com (S.-Y.P.); 4 Department of Veterinary Surgery, College of Veterinary Medicine, Gyeongsang National University, Jinju-si 52828, Gyeongsangnam-do, Republic of Korea; ch.moon@gnu.ac.kr

**Keywords:** cat, tracheal lymphoma, B-cell lymphoma, tracheal obstruction, tracheal resection and anastomosis, cross-field ventilation, CHOP chemotherapy, case report

## Abstract

Intratracheal lymphoma is an uncommon cause of tracheal obstruction in cats. A 10-year-old spayed female Korean Shorthair cat was referred after 1 year of progressive respiratory signs and presented in lateral recumbency with open-mouth breathing and stridor. Non-contrast whole-body computed tomography (CT) without general anesthesia showed a 15.99 × 8.81 × 7.05 mm intraluminal mass in the cervical trachea that reduced luminal cross-sectional area by 87%; no other mass lesion was identified in the scanned field. Hematocrit and total thyroxine were within reference intervals, and N-terminal pro-B-type natriuretic peptide was mildly elevated. As respiratory effort persisted despite overnight oxygen, four tracheal rings were resected with end-to-end anastomosis the next day using cross-field ventilation through a temporary tracheotomy. Histopathology and immunohistochemistry identified a PAX5-positive B-cell lymphoma with clear but narrow margins. Postoperative feline leukemia virus and feline immunodeficiency virus tests were negative. Two CHOP-based cycles were given. Systemic staging was incomplete and follow-up imaging was limited to 6.5 months, when CT showed no recurrence in the field examined. Tracheal lymphoma should be considered in cats with an obstructing tracheal mass; emergency resection relieved the obstruction here, but one case cannot define the optimal treatment sequence.

## Simple Summary

Lymphoma is a common cancer in cats; however, a tumor growing inside the windpipe (trachea) is uncommon. We describe a 10-year-old cat that was referred because of long-standing noisy breathing that had become severe. The cat was breathing with its mouth open and lying on its side. Computed tomography, performed under light sedation without general anesthesia, showed a mass blocking nearly 90% of the windpipe. Because breathing did not improve with oxygen and supportive care overnight, the affected segment of the windpipe was removed the next day and the two ends were re-joined. The tumor was a B-cell lymphoma. A shortened course of chemotherapy followed, and the cat had no sign of the tumor at the site of surgery 6.5 months later. Because the cat was not fully staged for lymphoma elsewhere in the body and was followed for a limited time, this report describes what was done and observed in one patient instead of a general rule for treating such cats.

## 1. Introduction

Lymphoma is among the most frequently diagnosed hematopoietic neoplasms in cats, yet primary laryngeal or tracheal involvement remains an uncommon extranodal presentation [1,2,3,4,5]. In a retrospective series of 23 cats with laryngeal or tracheal lymphoma, most tumors had a B-cell phenotype, and prolonged survival was reported in a subset of patients after surgical or medical treatment [1]. Long-term survival has also been documented in cats with lymphoma [6]. Laryngeal and tracheal masses typically cause non-specific signs of upper airway obstruction (dyspnea, stridor, wheezing, coughing, and open-mouth breathing) that must be distinguished from feline asthma and inflammatory or infectious airway disease [3,4,7,8]. Because a substantial proportion of feline laryngotracheal masses are neoplastic, neoplasia should be prioritized among the differentials [4,9,10].

Tracheal lymphoma is generally chemoresponsive. Systemic chemotherapy has produced radiographic tumor reduction and clinical remission in cats with intratracheal lymphosarcoma [1,11], and tracheal resection with anastomosis followed by chemotherapy has yielded a favorable long-term outcome [12]. Surgical resection is likewise the mainstay of treatment for primary tracheal tumors in human medicine [13]. In the published feline reports, the diagnostic work-up, including endoscopy or advanced imaging under general anesthesia, could be completed before treatment was planned [11,12,14]; how such a work-up should be modified when a cat presents with severe obstruction has received little attention [1,10,11,12]. Rib fractures in cats are usually traumatic, although non-traumatic fractures have been described in cats with respiratory disease, and repetitive forceful respiratory effort or coughing has been proposed as a contributing mechanism [15,16,17,18].

This report describes a cat with intratracheal B-cell lymphoma that presented with severe tracheal obstruction and was managed by emergency tracheal resection and anastomosis followed by an abbreviated adjuvant chemotherapy protocol. The aim was to document the clinical course, the diagnostic and therapeutic steps taken under the constraints of the respiratory status of the patient, and the limitations of the data, in accordance with the CARE guidelines for case reports.

## 2. Case Presentation

### 2.1. Patient Information and History

A 10-year-old spayed female Korean Shorthair cat (4.0 kg) was referred on 13 November 2024, for evaluation of a suspected intratracheal mass. The owner reported progressive respiratory signs for approximately 1 year, with marked worsening over the preceding 5 months. Thoracic radiography performed at a primary-care clinic in June 2024 first raised suspicion of an intraluminal tracheal mass; however, no additional diagnostic imaging was performed before referral. Prior to referral, the cat had received symptomatic treatment for suspected upper respiratory tract disease at the primary-care clinic, including a bronchodilator (theophylline) and mucolytic agent (N-acetylcysteine). No other underlying disease requiring ongoing treatment was reported. The cat was kept exclusively indoors as the only cat in the household, was fed a commercial dry diet, and was up to date on routine vaccination and deworming. Appetite was maintained throughout, and no vomiting or altered defecation was reported.

### 2.2. Clinical Findings at Presentation

The cat presented at 17:09 in lateral recumbency with open-mouth breathing, increased respiratory effort, stridor, and wheezing. The respiratory rate was 48 breaths/min, the heart rate was 190 beats/min, and the rectal temperature was 40.2 °C. Blood pressure, pulse oximetry, mucous membrane color, and peripheral lymph node palpation were not documented in the admission record and are thus not reported. Thoracic radiographs obtained at the referring clinic on the day of referral showed an intratracheal soft-tissue opacity without evidence of rib fracture. Bloodwork performed at the referring clinic prior to referral showed a hematocrit of 44.8% (reference interval [RI], 30.3–52.3%), a mildly elevated N-terminal pro-B-type natriuretic peptide concentration of 141.5 pmol/L (RI, 0–100 pmol/L), and a total thyroxine concentration of 1.5 µg/dL (RI, 0.8–4.7 µg/dL). Blood lactate concentration was 6.17 mmol/L (RI, 0.5–2.0 mmol/L) in a venous sample collected at the referring clinic under routine physical restraint and prior to oxygen supplementation; lactate was not re-measured after referral. The remaining hematological and serum biochemical variables were within their reference intervals. A blood smear review and leukocyte morphology were not documented. The clinical picture was attributed to tracheal obstruction instead of primary cardiac disease. Feline leukemia virus (FeLV) antigen and feline immunodeficiency virus (FIV) antibody testing was not performed at presentation; testing was performed during postoperative hospitalization (see Section 2.5).

### 2.3. Diagnostic Assessment

A 24-gauge intravenous catheter was placed in a forelimb, and butorphanol (0.2 mg/kg IV) was administered immediately prior to imaging. Non-contrast whole-body computed tomography (CT) from the head to the distal extremities was performed at 17:23 without general anesthesia (Philips Access CT 16; Philips Healthcare, Cleveland, OH, USA). The cat was placed in a fabric carrier, and towels were used to fill the surrounding space to limit movement. Oxygen from the anesthetic ventilator was delivered near the nose immediately before and after acquisition, which lasted approximately 15 s. Because of the short acquisition time, instrumental monitoring was not used during the scan; the respiratory pattern was observed directly by the staff before and after the scan. In addition to the veterinarian operating the scanner, one veterinarian and one veterinary technician were present with intubation equipment prepared for emergency airway management. Acquisition parameters were 120 kVp, 110 mAs, 0.75 s gantry rotation, pitch 1.0625, and 16 × 0.8 mm collimation; images were reconstructed with the iterative algorithm of the vendor (iDose^4^, level 3) at 1.0 mm slice thickness and 1.0 mm increment on a 512 × 512 matrix using soft-tissue and high-resolution lung kernels, and axial, sagittal, and dorsal reconstructions were reviewed.

CT confirmed an intraluminal soft-tissue-attenuating mass within the cervical trachea at the level of the third cervical vertebra, appearing to arise from the dorsal membranous wall (Figure 1b–e). The mass measured 15.99 × 8.81 × 7.05 mm (length × width × height), and the longitudinal extent of tracheal involvement was 15.99 mm. The minimum residual luminal diameter at the most severely obstructed level was 1.55 mm. The residual luminal cross-sectional area (CSA) at this level was 8.84 mm^2^, compared with a reference CSA of 67.78 mm^2^ in the adjacent normal trachea. Using the formula [1 − (CSA_stenotic_/CSA_reference_)] × 100, the estimated luminal obstruction was approximately 87%. Luminal diameters and CSA were measured on transverse images with the cursors placed on the inner tracheal wall, as described for CT measurement of the feline trachea [19]. The lesion had a broad-based attachment to one side of the tracheal wall and protruded into the lumen without circumferential wall contact, and no extraluminal extension was identified. No overt regional lymphadenopathy was seen, although assessment of the regional lymph nodes was limited by the absence of contrast enhancement. No other mass lesion or lymphadenomegaly was identified within the scanned field; however, non-contrast CT is not an adequate substitute for formal lymphoma staging (see Section 3.4). The location of the mass within the surgically accessible cervical trachea and the limited length of involvement were considered compatible with segmental resection and end-to-end anastomosis.

After CT, the cat was hospitalized in an oxygen cage with continuous oxygen supplementation and was monitored clinically overnight; butorphanol (0.2 mg/kg IV) was administered once, at 09:00 on November 14, for sedation. Surgery was not performed immediately because the attending clinicians elected to assess whether the cat would stabilize with oxygen and supportive care alone. During the night, the respiratory rate was 36 breaths/min with persistent increased effort, the heart rate was 180 beats/min, and the rectal temperature was 40.0 °C. Because respiratory effort did not improve, surgical relief of the obstruction was elected on November 14. A right lateral thoracic radiograph obtained at 21:15 that day confirmed the tracheal mass and, in addition, showed multiple rib fractures (Figure 1a) that had not been present on the radiographs by the referring clinic of November 13. The fractures involved the mid-shaft of the right sixth to thirteenth ribs; those of the seventh to thirteenth ribs were displaced, with the fragments directed cranioventrally in the seventh to ninth ribs and caudodorsally in the tenth to thirteenth ribs, and the fracture margins were sharp without evidence of healing, consistent with acute fractures. Therefore, fractures developed between the two examinations, but their cause could not be established from the available images, and handling-related injury could not be excluded; they were recorded as fractures of uncertain origin. Anesthesia was induced at 21:45, and surgery began at 21:55, approximately 28 h after presentation.

### 2.4. Anesthesia and Surgical Treatment

Cefazolin (22 mg/kg IV) and famotidine (0.5 mg/kg IV) were administered prior to induction. Preoxygenation was provided by delivering oxygen near the nose until induction. Butorphanol (0.2 mg/kg IV) and medetomidine (10 µg/kg IV) were administered through the existing catheter, followed by alfaxalone in 0.5 mg/kg increments to a cumulative dose of 2 mg/kg IV. Orotracheal intubation was not attempted; spontaneous ventilation was maintained, and the cervical trachea was approached under injectable anesthesia until direct airway access was obtained. During this initial phase, pulse oximetry remained at or above 95%, oscillometric systolic arterial pressure was 90–100 mmHg, heart rate was 140–150 beats/min, and rectal temperature was 38.8–39.7 °C; end-tidal carbon dioxide (EtCO_2_) could not be measured before intubation.

Through a ventral cervical midline approach, the cervical trachea was exposed (Figure 2a). The lesion had been localized on preoperative CT and radiography to the level of the third cervical vertebra, with its base on the dorsal membranous wall, and its position was confirmed by palpation of the tracheal wall (Figure 2b); the exact ring numbers were not recorded and could not be determined retrospectively. A temporary tracheotomy was made between tracheal rings caudal to the lesion, separate from the planned resection margins, and a 3.0-mm endotracheal tube was inserted through it into the caudal tracheal lumen for cross-field ventilation (Figure 2c). Anesthesia was thereafter maintained with isoflurane in oxygen through a sterile circuit; EtCO_2_ was 37–42 mmHg and pulse oximetry remained at or above 97%. Four tracheal rings containing the mass, corresponding to approximately 16 mm and encompassing the 15.99-mm length of involvement identified on CT, were resected (Figure 2f,g). The extent of resection was limited to the mass-bearing segment identified on imaging and palpation to avoid unnecessary shortening of the trachea. The resected segment corresponded to approximately 9.7% of the total tracheal length measured on CT (164.69 mm), which is within the conservative limit provided for cats [14]. Frozen-section evaluation was not available. Prior to anastomosis, the cranial and caudal segments were manually extended and approximated to confirm that they could be apposed without tension.

End-to-end anastomosis was performed with 5-0 polydioxanone in a simple interrupted pattern, with sutures passed through the tracheal cartilage at approximately 1–2 mm intervals and knots placed extraluminally (Figure 2d,e); the exact number of sutures was not recorded. Four far–near–near–far sutures, with the far bites passed through the annular ligaments, were then added to reinforce the anastomosis and relieve tension. A second 3.0-mm endotracheal tube was placed orotracheally with its tip cranial to the anastomosis, and a leak test was performed by manual positive-pressure ventilation while saline was applied over the anastomosis; no air leakage was detected. The cross-field tube was then removed, and ventilation continued through the orotracheal tube. The temporary tracheotomy was not sutured, and the surrounding soft tissues were closed over it. The total durations of anesthesia, surgery, and cross-field ventilation were approximately 2 h 20 min, 1 h 50 min, and 1 h 10 min, respectively. Intubation was performed twice (cross-field and orotracheal). No episode of apnea, hypoxemia, hypotension, or arrhythmia was recorded during anesthesia.

### 2.5. Postoperative Care and Hospitalization

The cat was hospitalized from 13–23 November 2024. Intravenous fluid therapy (Plasma Solution A), cefazolin (22 mg/kg IV q12h), marbofloxacin (3 mg/kg SC q24h), and famotidine (0.5 mg/kg IV q12h) were started on November 14; the antimicrobials were administered empirically as perioperative prophylaxis, and famotidine was administered for gastric protection during multidrug therapy. Butorphanol (0.2 mg/kg IV q12h) was administered for analgesia from November 15 to 22. Blood was not sampled on November 14 or 15. On November 16, feline serum amyloid A (fSAA) was markedly elevated (135.6 µg/mL; RI, 0–5 µg/mL) with a white blood cell (WBC) count within the reference interval (11.89 × 10^9^/L; RI, 2.87–17.02 × 10^9^/L), and the rectal temperature reached 40.1 °C. On November 17, fSAA was 124.3 µg/mL and the WBC count 10.51 × 10^9^/L, with temperatures of 39.1–39.5 °C. On November 18, leukocytosis developed (WBC 17.44 × 10^9^/L) while fSAA remained elevated (92.0 µg/mL) and temperatures were 38.6–39.4 °C; clindamycin (11 mg/kg IV q12h) was added empirically on that day. Bacterial culture was not performed, and no clinical or radiographic evidence of aspiration pneumonia or surgical-site infection was noted; the escalation was based on the inflammatory markers alone, and infection was not microbiologically confirmed. The WBC count peaked at 19.75 × 10^9^/L on November 20 and returned to the reference interval by November 22 (14.59 × 10^9^/L), while fSAA declined progressively to 61.3 µg/mL on November 22. All injectable medications were administered through November 22; at discharge on November 23, the same drugs were continued orally at the same doses. At outpatient re-examinations, fSAA was 5.8 µg/mL with a WBC count of 7.62 × 10^9^/L on November 25, and below 5 µg/mL with a WBC count of 4.65 × 10^9^/L on November 30, when all oral medications, including the antimicrobials, were discontinued.

Nutrition was provided through a nasoesophageal tube from November 16 to 23, and the tube was removed when spontaneous food intake was confirmed. A neck brace was applied from the day after surgery and maintained for approximately 2 months; cervical flexion was further limited by restricting jumping and raising the food bowl, and the owner was instructed accordingly. During hospitalization, the respiratory pattern was monitored and no coughing was observed; swallowing was assessed when water was provided approximately 1 week after surgery, and no dysphagia was noted. Thoracic auscultation and serial thoracic radiographs showed no evidence of aspiration. Mild subcutaneous emphysema present immediately after surgery (Figure 3), attributable to the unsutured tracheotomy, resolved progressively on serial radiographs, and no clinical or radiographic sign of anastomotic dehiscence was identified. Laryngeal function and anastomotic stenosis were not assessed with dedicated testing. FeLV antigen and FIV antibody testing with a commercial rapid kit (Rapid FIV Ab/FeLV Ag Test Kit; Bionote, Hwaseong, Republic of Korea) performed during hospitalization was negative for both. The cat was discharged on November 23 once respiratory status and appetite had normalized and spontaneous food intake was confirmed.

### 2.6. Histopathology and Immunohistochemistry

The resected segment was submitted to a commercial reference laboratory, and evaluation was performed by a board-certified veterinary pathologist (Diplomate, American College of Veterinary Pathologists); the slides were not reviewed by a second pathologist. On a transverse section, a broad-based, well-circumscribed, ovoid, densely cellular neoplasm measuring just under 0.7 cm in diameter at the level of the histologic cross-section (not the craniocaudal length) arose from the luminal mucosa and extended into the lumen (Figure 4a). The tumor consisted of sheets of mononuclear cells with scant to moderate amphophilic cytoplasm and polyhedral nuclei with clumped chromatin and one or more nucleoli, with anisocytosis and anisokaryosis and a mitotic count of 10 per 2.37 mm^2^ (Figure 4b). The tumor originated in the region of the non-cartilaginous tracheal membrane and focally abutted, but did not infiltrate, the trachealis muscle; angiolymphatic invasion was not detected. Immunohistochemistry showed intense nuclear PAX5 labeling in more than 95% of the neoplastic population and CD3 labeling in fewer than 5% of cells (Figure 5). Surgical margins were reported as clear on gross and histologic assessment but narrow; the width of the narrowest margin could not be determined from the cross-section examined, and the specimen and slides are no longer available for additional measurement. Cell size classification, necrosis, and the counting method beyond the reported area were not described in the report and are not inferred here. Clonality testing was not recommended by the pathologist and was not performed, and no additional markers were applied. The diagnosis was tracheal B-cell lymphoma.

### 2.7. Adjuvant Chemotherapy

Histologically clear margins indicate local excision of the tracheal lesion but do not establish that the lymphoma was confined to the trachea. Because complete staging had not been performed and microscopic residual or systemic disease could not be excluded, adjuvant CHOP-based chemotherapy was started on 13 December 2024. An abbreviated two-cycle protocol was selected on the basis of the financial constraints of the owner and the assessment of the expected benefit and treatment burden by the attending clinician in a 10-year-old cat; the decision was not based on evidence that two cycles constitute adequate treatment for feline tracheal B-cell lymphoma. A complete blood count was obtained prior to each administration. Neutropenia was graded according to the Veterinary Cooperative Oncology Group common terminology criteria for adverse events, version 2 (VCOG-CTCAE v2) [20]. Chemotherapy was withheld for grade 2 or higher neutropenia, filgrastim (5 µg/kg SC; Leukostim, Dong-A ST, Seoul, Republic of Korea) was administered, and treatment was resumed when a repeat count showed recovery. The intended weekly schedule and the schedule actually delivered, with pre-treatment counts, are shown in Table 1.

The nadir neutrophil count was 0.83 × 10^9^/L. Grade 3 neutropenia was identified on 27 January and 20 February 2025, on each occasion leading to withholding of treatment and a single dose of filgrastim, with resumption after 3 and 2 days, respectively. On February 6, grade 1 neutropenia was present, but treatment was postponed for 7 days because of the preceding grade 3 event and the schedule of the owner, and the vincristine dose was reduced from 0.7 to 0.6 mg/m^2^. One further 2-day delay (January 18 to 20) was due to owner scheduling. Two cycles were completed on 22 February 2025, over approximately 10 weeks. No other adverse event was recorded.

### 2.8. Follow-Up and Outcome

Respiratory signs resolved after surgery, and the skin staples were removed after uncomplicated healing. The final documented follow-up was an in-hospital re-examination on 31 May 2025, approximately 6.5 months after surgery, comprising physical examination, owner interview, complete blood count, serum biochemistry, venous blood gas and electrolyte analysis, total thyroxine concentration, thoracic radiography, and whole-body contrast-enhanced CT; no clinically significant abnormality was identified on any of these tests. The owner reported no recurrence of respiratory signs and a general condition comparable to that prior to the onset of illness, and no further anticancer or supportive treatment had been provided after the second chemotherapy cycle. CT showed a patent cervical trachea without residual or recurrent mass at the anastomotic site and no evidence of regional invasion or of metastatic disease within the field examined (Figure 6). The follow-up period does not permit conclusions regarding long-term disease control.

### 2.9. Timeline

The chronological course of the case, distinguishing documented observations, clinical interpretations, and treatment decisions, is summarized in Figure 7.

## 3. Discussion

### 3.1. Clinical Decision-Making in This Case

This cat was referred with a long history of progressive respiratory signs and presented with open-mouth breathing, stridor, and lateral recumbency, which are recognized indicators of severe upper airway compromise that warrant urgent attention [21,22]. CT was obtained under light sedation because general anesthesia and orotracheal intubation were judged to carry a substantial risk with an unquantified obstruction; the scan documented a lesion that reduced the luminal cross-sectional area by approximately 87%. The decision to operate was not taken at that point. The cat was managed in an oxygen cage overnight, and surgery was elected only after respiratory effort persisted despite oxygen and supportive care. This sequence is worth stating plainly because the delay of approximately 28 h between presentation and surgery shows that the cat was not deteriorating minute by minute; instead, it remained dependent on supplemental oxygen with persistent effort, and the clinicians concluded that the obstruction was unlikely to resolve without mechanical relief. Chemotherapy can reduce tracheal lymphoma, but the response is not immediate [1,11], and the clinicians did not consider it likely that the obstruction could be managed medically in the interim. Whether the same decision would be appropriate in another cat depends on the position and resectability of the mass, the degree of obstruction, the expected time to a chemotherapeutic response, surgical access, and anesthetic risk.

Tracheal resection and anastomosis performed under these circumstances carries more risk than the same procedure performed electively. There was little opportunity to optimize the patient beforehand, and the margin for anesthetic decompensation was reduced. Anastomotic dehiscence is the most serious complication of the procedure in cats, and a fatal outcome after tracheal resection for lymphoma has been described [9]. In a recent series of 20 dogs and cats undergoing tracheal resection and anastomosis, complications were common but most animals survived to discharge [23].

### 3.2. Anesthetic and Ventilatory Management

Securing ventilation was the principal anesthetic challenge. With an intraluminal mass, orotracheal intubation may further narrow the airway or dislodge the tumor [24], and it was not attempted. Instead, the cervical trachea was approached with the cat breathing spontaneously under injectable anesthesia, and a temporary tracheotomy was made caudal to the lesion for cross-field ventilation. This interval, during which no capnography was available, was the period of greatest risk; pulse oximetry, blood pressure, and heart rate remained within acceptable limits, and once the cross-field tube was in place EtCO_2_ was 37–42 mmHg. Alfaxalone was chosen for induction because of its favorable cardiovascular profile at clinical doses and the ability to titrate it slowly to effect [25,26], and the recorded monitoring data in this cat were consistent with that expectation. Cross-field ventilation through a separate tracheotomy, instead of through the transected trachea itself, kept the tube away from the resection margins during suturing; a similar approach with a tube passed through the surgical site has been described in a cat undergoing resection for squamous cell carcinoma [14]. The tracheotomy was left unsutured, which is likely to have contributed to the transient subcutaneous emphysema observed postoperatively; whether closure of the tracheotomy would have been preferable cannot be determined from this case.

### 3.3. Rib Fractures of Uncertain Origin

Multiple rib fractures were identified on the radiograph obtained on 14 November 2024, and were absent on the radiographs of the referring clinic of the previous day. Therefore, serial images establish that the fractures developed within the roughly 28 h between the two examinations, but they do not establish how. Non-traumatic rib fractures associated with sustained respiratory effort or coughing have been described in cats and in people [15,16,17,18], and the markedly increased work of breathing by the cat over this period is one plausible explanation. However, no direct evidence links the fractures to respiratory effort, and injury during transport, restraint, positioning for CT, or handling in the oxygen cage cannot be excluded. The fractures were not used as an indicator of respiratory workload in the decision to operate, and they are reported here as an incidental finding of uncertain cause.

### 3.4. Adjuvant Chemotherapy and Limitations of Staging

Lymphoma is a systemic disease, and multi-agent protocols over several months are the usual standard in cats [27,28,29,30,31]. Adjuvant chemotherapy after surgical excision of a discrete lymphoma mass has been reported in cats with gastrointestinal lymphoma [32,33,34], but there is no evidence that two cycles of a CHOP-based protocol constitute adequate treatment for feline tracheal B-cell lymphoma, and the favorable status of this cat at 6.5 months cannot be taken as validation of the abbreviated regimen.

The two-cycle protocol was chosen because of the financial constraints of the owner and the weighing of the expected benefit against the burden of treatment in an older cat by the clinician [35]; even this shortened course produced two episodes of grade 3 neutropenia requiring filgrastim and a dose reduction, which is consistent with the toxicity reported for multi-agent protocols in cats [36].

Staging was incomplete, and this limitation was not confined to the emergency period. Abdominal ultrasonography was not performed after the cat had stabilized, and the postoperative FeLV and FIV tests, although negative, were the only staging-related tests added after surgery. The preoperative non-contrast whole-body CT included the abdomen and showed no lymphadenomegaly or mass lesion, but non-contrast CT has limited sensitivity for abdominal lymphoma and does not replace ultrasonography with cytology, and the follow-up CT cannot exclude disease outside the scanned field. Accordingly, the lymphoma in this cat should be described as tracheal B-cell lymphoma instead of as a primary or localized tumor, and the histologically clear, narrow margins should be understood as evidence of complete gross excision of the tracheal lesion, not of the absence of systemic disease. A full staging work-up once such a patient is stable would be advisable and would have strengthened both the interpretation of the tumor and the choice of chemotherapy.

### 3.5. Comparison with Previous Reports

Table 2 compares the present case with published cats that underwent tracheal resection and anastomosis or medical treatment for tracheal lymphoma or other tracheal tumors, using the information actually reported in each paper. The Siamese cat described by Bataller et al. (2017) was bright and responsive at presentation with tachypnea (50–60 breaths/min) and mild inspiratory dyspnea, tracheoscopy showed a residual lumen of 4 mm, FeLV and FIV were negative, abdominal staging was declined for financial reasons, seven rings were resected with incomplete margins, and a 6-week COP protocol was followed by no recurrence on tracheoscopy at 20 months [12]. The cat reported by Miller et al. (2020) underwent resection of five rings (approximately 3 cm) for squamous cell carcinoma with incomplete margins and cross-field ventilation through the surgical site; no adjuvant treatment was pursued, and the cat was well at 120 days [14]. Dugas et al. (2011) documented radiographic regression of an intratracheal lymphosarcoma after systemic chemotherapy without surgery [11], and Kanemoto et al. (2023) described a cat that died 7 days after tracheal resection for lymphoma from suspected anastomotic dehiscence [9]; neither report provides enough detail on the respiratory status at presentation for comparison. The present cat differs from those reported by Bataller and Miller primarily in the severity of obstruction at presentation (lateral recumbency and open-mouth breathing, residual luminal diameter 1.55 mm, and approximately 87% obstruction by cross-sectional area), which precluded endoscopy or contrast CT under anesthesia prior to surgery as well as in the chemotherapy approach. It resembles the Bataller cat in that staging remained incomplete for financial reasons. The follow-up in the present case (6.5 months by CT) is shorter than in the Bataller cat (20 months by tracheoscopy), so the outcomes are not directly comparable.

The strengths of this report are the documented perioperative and chemotherapy records, including times, monitoring values, and pre-treatment blood counts, and the serial imaging that dates the rib fractures. Its limitations are those of a single retrospectively documented case: several items were not recorded at the time (admission blood pressure and pulse oximetry, mucous membranes, lymph node palpation, exact ring numbers, suture count, and margin width), staging was incomplete, and follow-up was limited to 6.5 months, so recurrence-free interval, progression-free interval, and survival cannot be inferred beyond the observation period. The report was prepared in accordance with the CARE guidelines, and a completed checklist is provided as supplementary material [37].

## 4. Conclusions

In this cat, emergency tracheal resection and anastomosis was technically feasible and relieved severe tracheal obstruction caused by B-cell lymphoma, and the cat was free of recurrence at the surgical site based on CT 6.5 months later. Tracheal lymphoma should be included in the differential diagnosis of an obstructing tracheal mass in cats. Emergency surgical relief may be considered when obstruction causes immediate danger and less invasive stabilization is unlikely to maintain airway patency; however, this single case, with incomplete staging and limited follow-up, cannot establish that surgery should generally precede chemotherapy or that an abbreviated chemotherapy protocol is adequate.

## Figures and Tables

**Figure 1:**
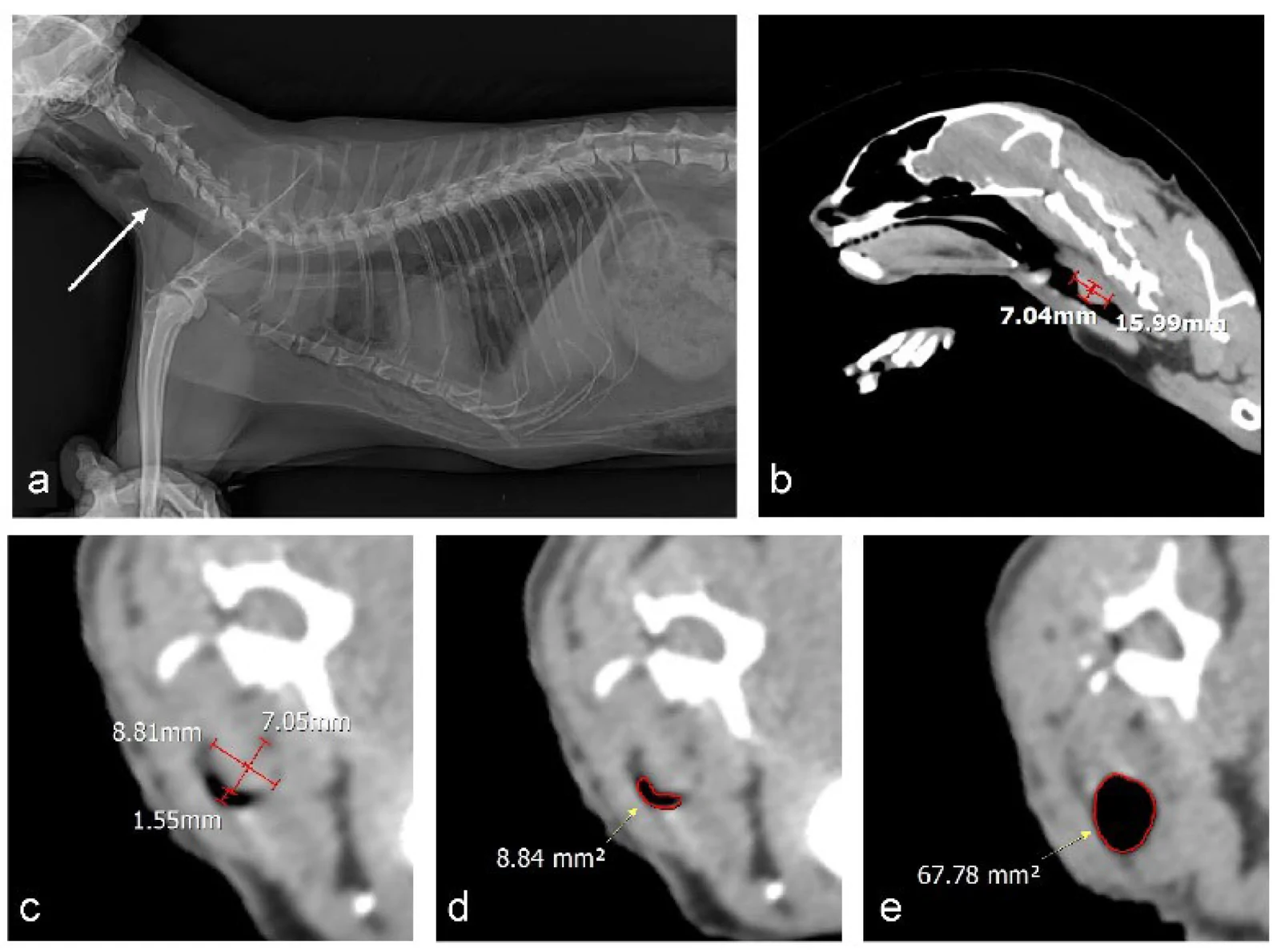
Diagnostic imaging in a cat with tracheal obstruction caused by an intraluminal tracheal mass. (**a**) Right lateral thoracic radiograph obtained on 14 November 2024, showing a focal intraluminal soft-tissue opacity (arrow) with marked narrowing of the cervical tracheal lumen; multiple rib fractures of uncertain origin are also present. (**b**–**e**) Non-contrast CT images (soft-tissue window) obtained at presentation without general anesthesia after intravenous butorphanol. (**b**) Sagittal reconstruction: the intraluminal mass at the level of C3 measures 15.99 mm in length and 7.04 mm in height. (**c**) Transverse image at the level of the mass: width 8.81 mm, height 7.05 mm, and minimum residual luminal diameter 1.55 mm. (**d**) Transverse image at the most severely obstructed level with the residual luminal cross-sectional area (CSA) outlined in red (8.84 mm^2^). (**e**) Transverse image of the adjacent normal trachea with the reference luminal CSA outlined in red (67.78 mm^2^). Estimated obstruction = [1 − (CSA_stenotic_/CSA_reference_)] × 100 ≈ 87%.

**Figure 2:**
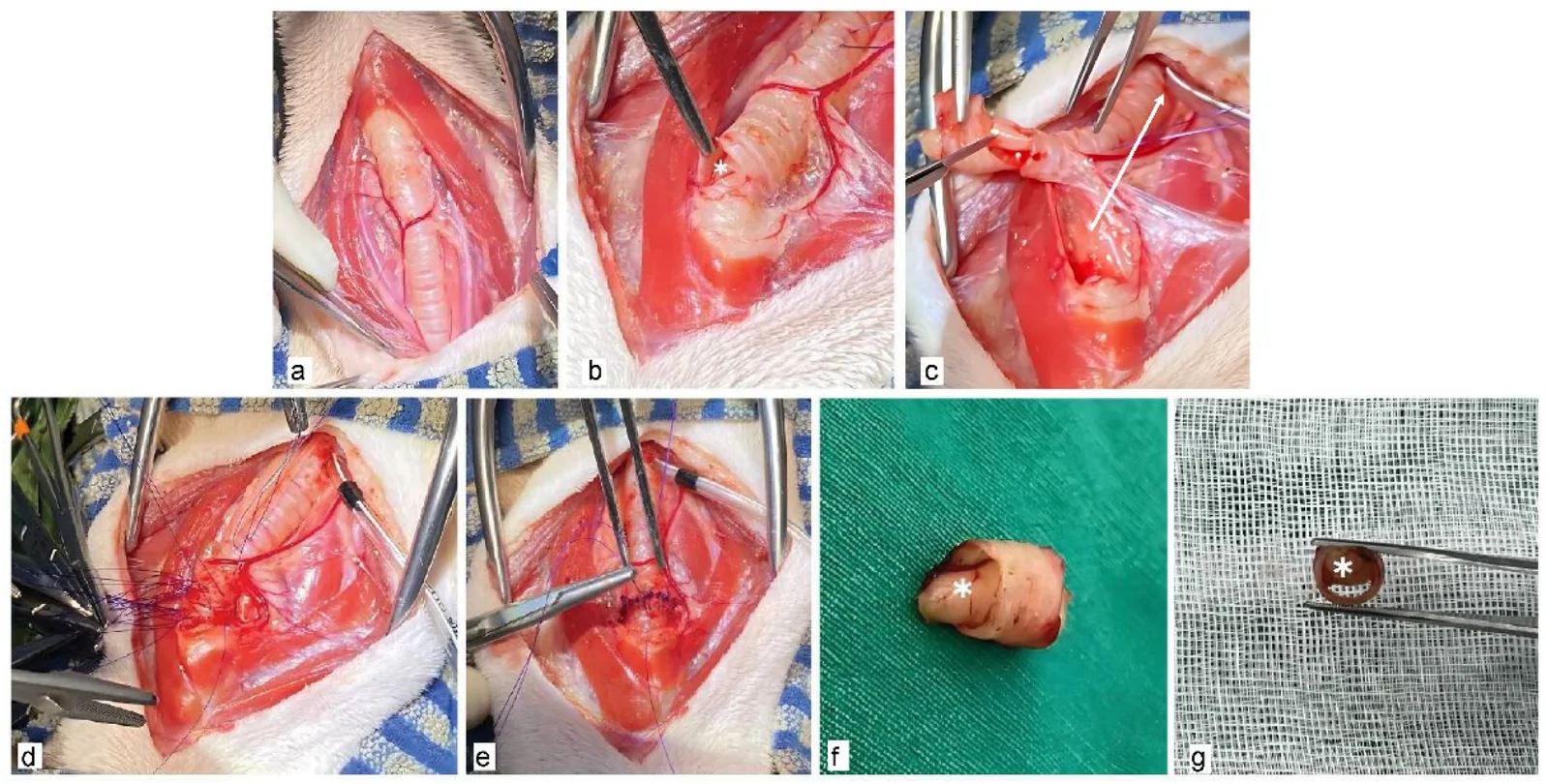
Intraoperative resection of a tracheal mass (asterisk) and end-to-end anastomosis. (**a**) Ventral cervical exposure of the trachea. (**b**) Identification of the intraluminal mass after isolation of the affected segment. (**c**) Temporary tracheotomy caudal to the lesion with a 3.0-mm endotracheal tube (arrow) inserted into the caudal tracheal lumen for cross-field ventilation. (**d**,**e**) End-to-end anastomosis with simple interrupted 5-0 polydioxanone sutures. (**f**,**g**) Resected segment containing the intraluminal mass (asterisk); the cross-section shows marked luminal narrowing.

**Figure 3:**
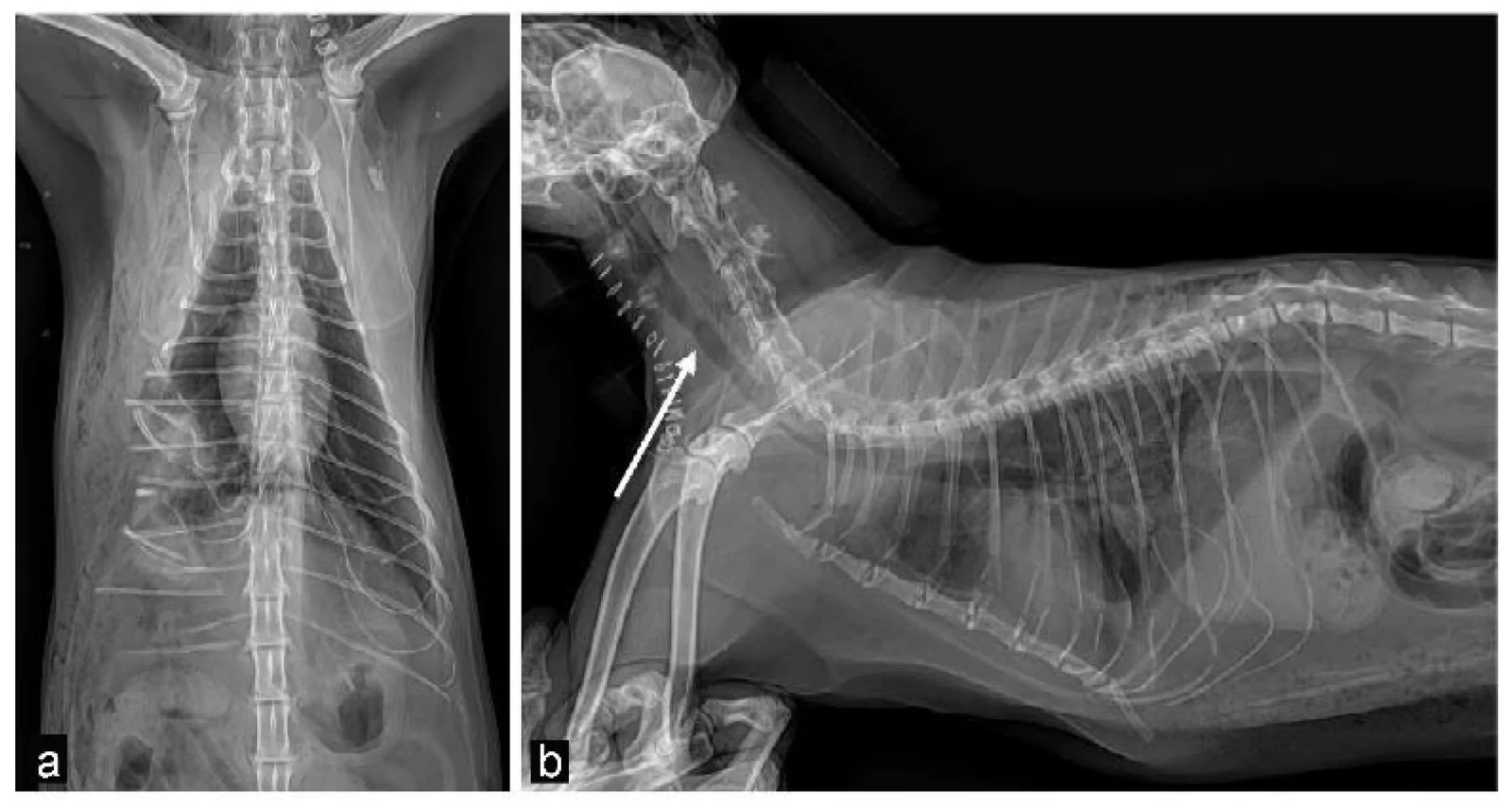
Postoperative thoracic radiographs. (**a**) Ventrodorsal and (**b**) right lateral views show restoration of the tracheal lumen (arrow) after removal of the intraluminal mass, with subcutaneous emphysema along the cervical and cranial thoracic body wall and no residual tracheal narrowing.

**Figure 4:**
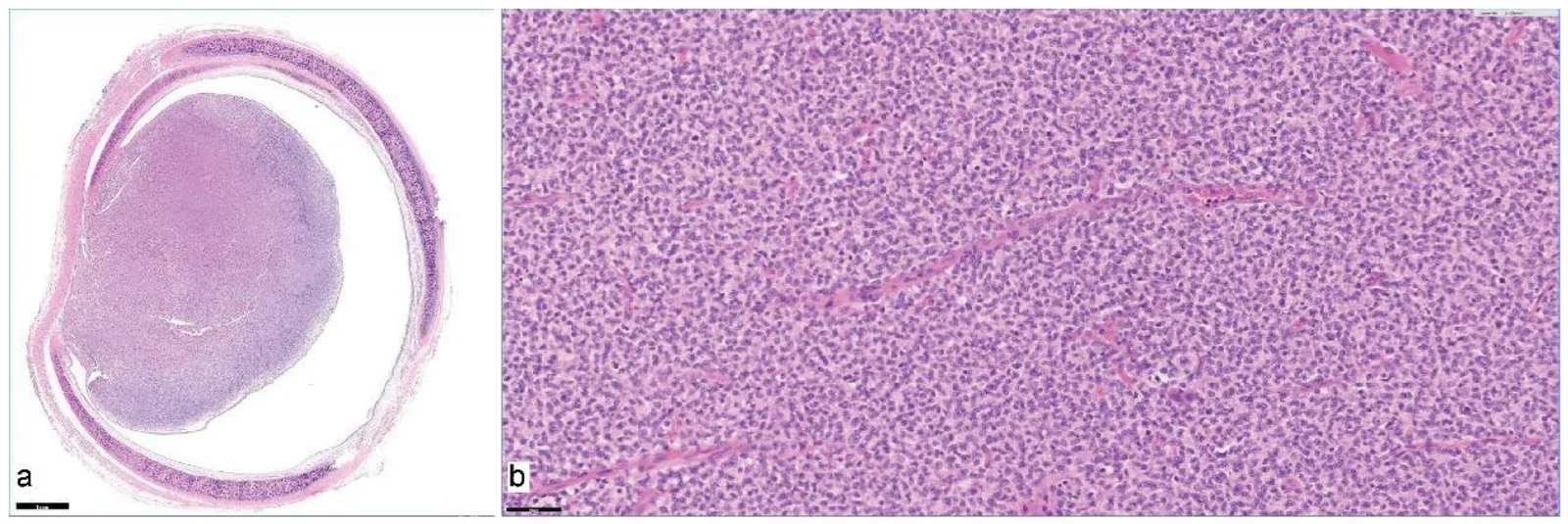
Histopathological features of the resected intratracheal mass. (**a**) 10×, H&E: transverse section of the resected trachea showing a well-circumscribed, broad-based intraluminal mass with marked luminal obstruction (scale bar = 1 mm). (**b**) 400×, H&E: densely packed round neoplastic cells with scant cytoplasm, anisokaryosis, and mitotic figures, consistent with lymphoma (scale bar = 50 µm).

**Figure 5:**
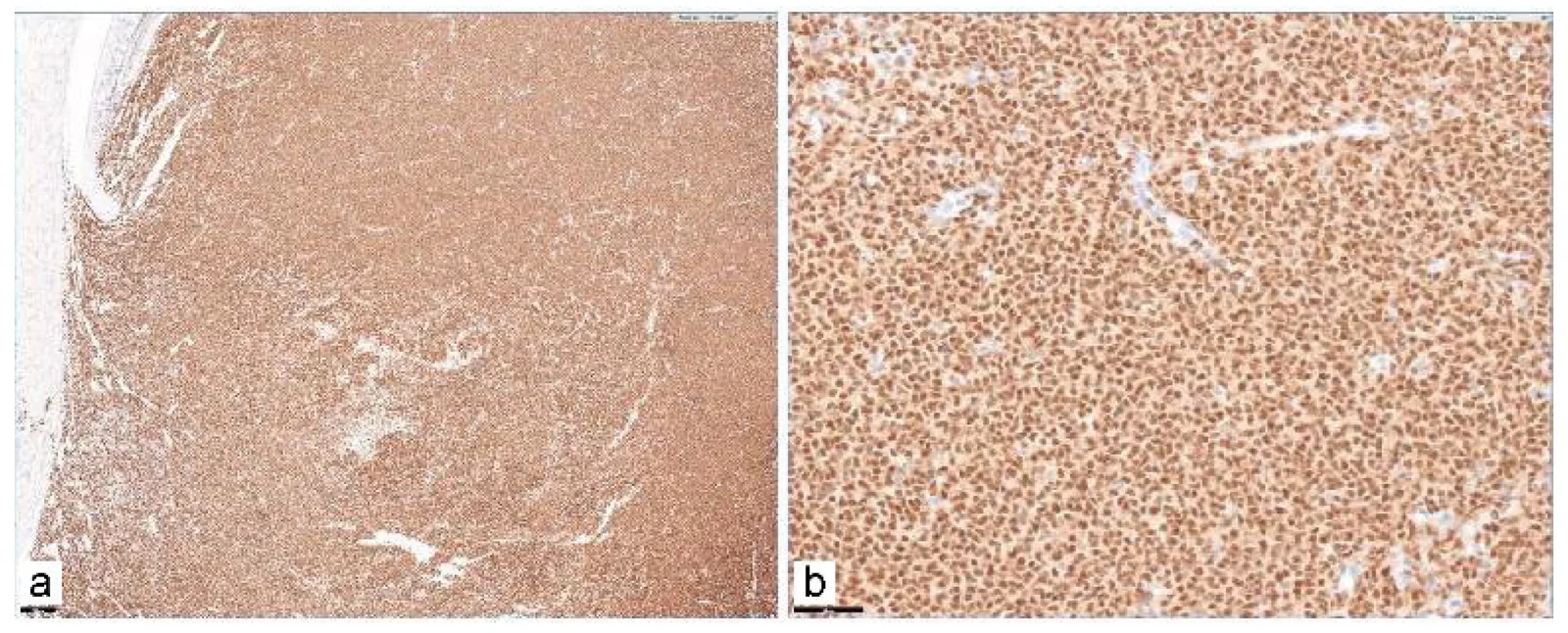
Immunohistochemical staining for PAX5, showing diffuse strong nuclear immunoreactivity throughout the neoplastic lymphoid population, consistent with a B-cell immunophenotype. (**a**) 50×, PAX5 IHC (scale bar = 200 µm). (**b**) 400×, PAX5 IHC (scale bar = 50 µm).

**Figure 6:**
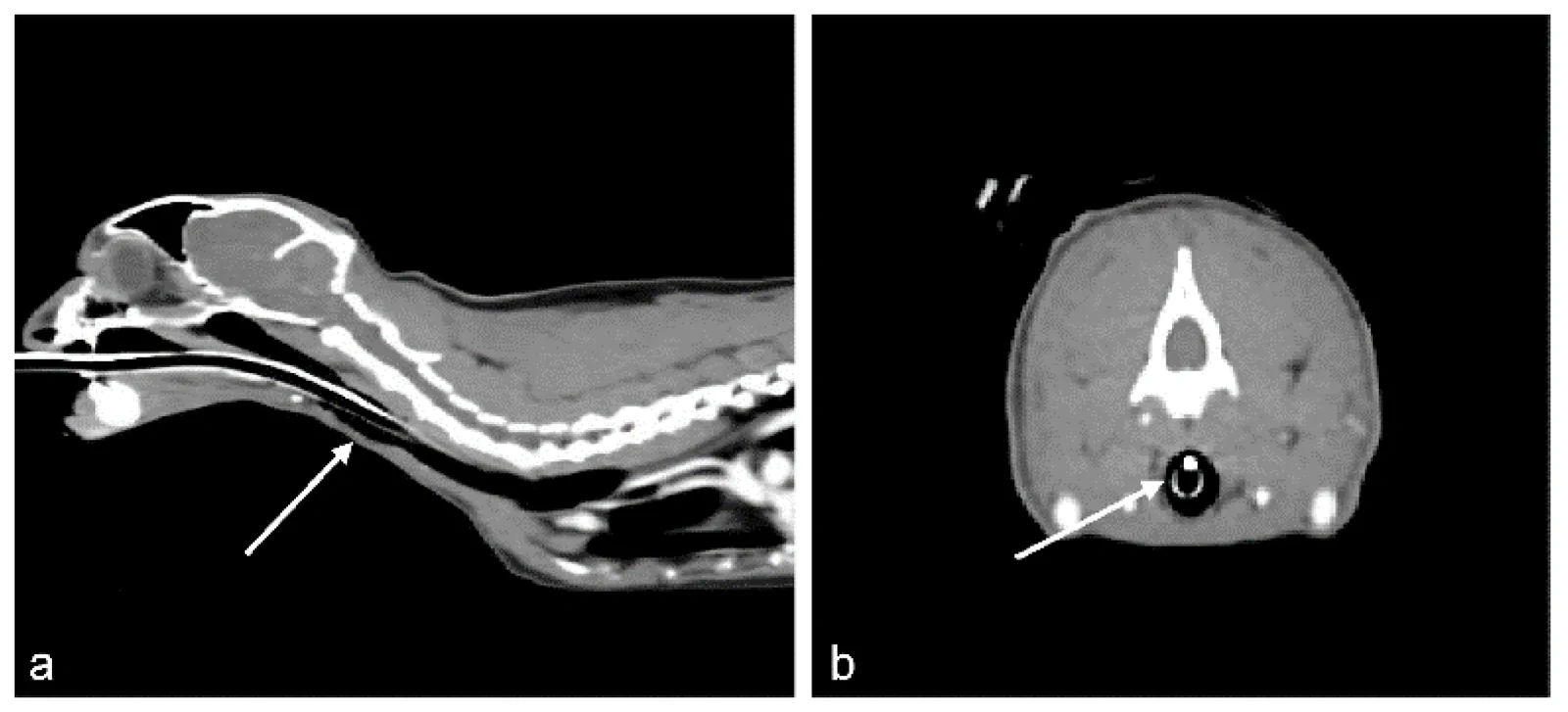
Follow-up contrast-enhanced CT acquired approximately 6.5 months after surgical resection of the intratracheal lymphoma. (**a**) Sagittal image showing a patent cervical tracheal lumen with no evidence of a recurrent intraluminal mass (arrow). (**b**) Transverse image at the previous lesion site, demonstrating preservation of the tracheal airway diameter, with no residual or recurrent soft-tissue-attenuating mass (arrow) and no evidence of regional invasion.

**Figure 7:**
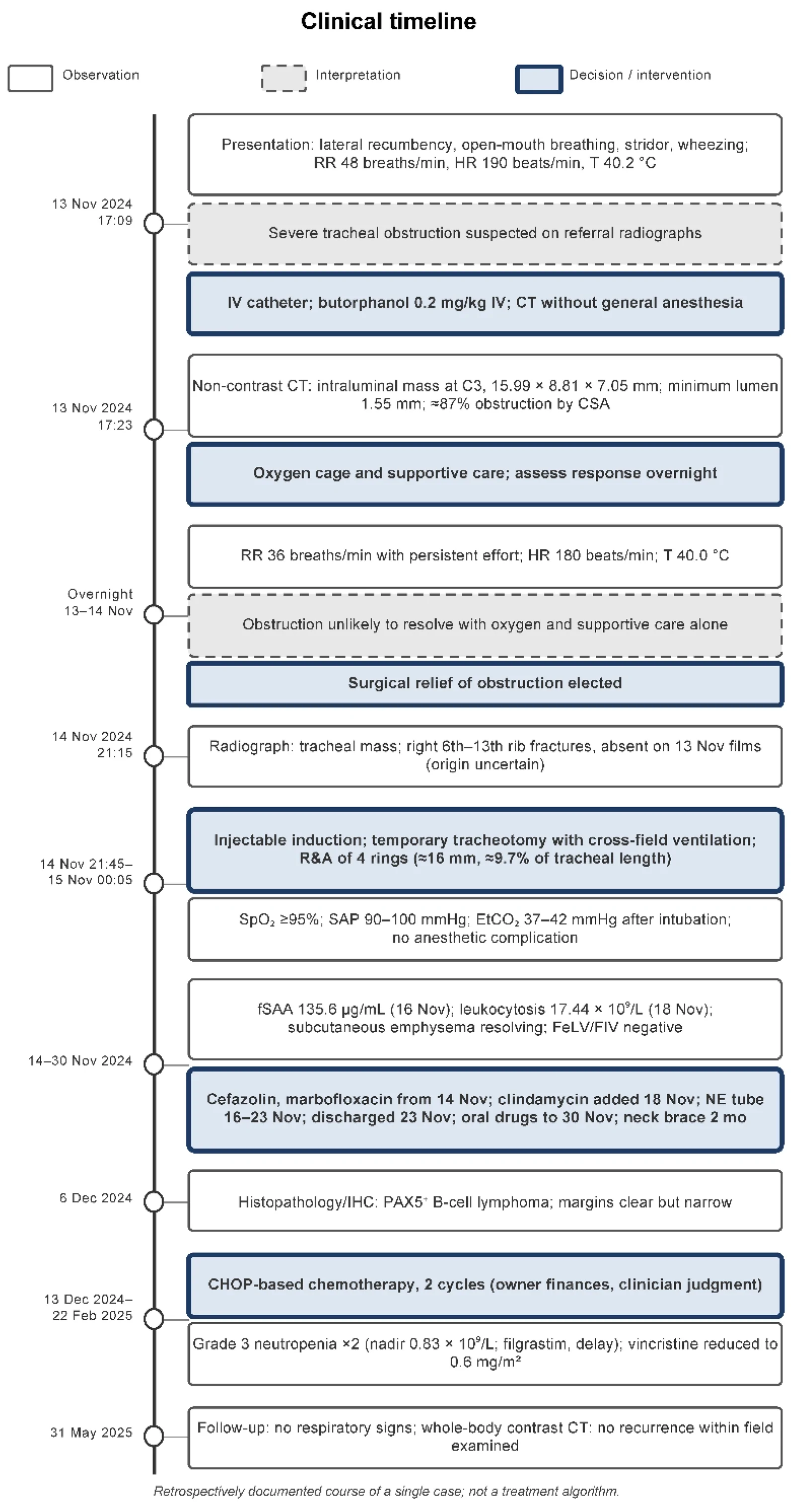
Clinical timeline of the case. Documented observations (clinical signs, imaging, laboratory, and pathology findings), clinical interpretations, and diagnostic or therapeutic decisions are shown as visually distinct elements. The timeline represents the retrospectively documented course of this single case and is not intended as a general treatment algorithm or a validated management pathway. CT, computed tomography; R&A, resection and anastomosis; CHOP, cyclophosphamide, doxorubicin, vincristine, and prednisolone.

**Table 1:** Intended and delivered schedule of the CHOP-based adjuvant chemotherapy, with pre-treatment blood counts and neutropenia grade.

Treatment	Intended Date	Actual Date	Agents and Doses	WBC/Neutrophils Prior to Treatment (×10^9^/L) a	Neutropenia Grade b	Action
Cycle 1, wk 1	13 Dec 2024	13 Dec 2024	Vincristine 0.7 mg/m^2^ IV; prednisolone 1 mg/kg PO q12h × 7 d	8.58/6.11	–	As scheduled
wk 2	20 Dec 2024	20 Dec 2024	Cyclophosphamide 250 mg/m^2^ IV; prednisolone 0.75 mg/kg PO q12h × 7 d	7.10/2.62	–	As scheduled
wk 3	27 Dec 2024	27 Dec 2024	Vincristine 0.7 mg/m^2^ IV; prednisolone 0.5 mg/kg PO q12h × 7 d	6.81/5.90	–	As scheduled
wk 4	4 Jan 2025	4 Jan 2025	Doxorubicin 1 mg/kg IV; prednisolone 0.5 mg/kg PO q12h × 7 d	7.18/5.04	–	As scheduled
wk 5	–	–	Rest week	–	–	–
Cycle 2, wk 6	18 Jan 2025	20 Jan 2025	Vincristine 0.7 mg/m^2^ IV; prednisolone 0.5 mg/kg PO q24h × 7 d	4.60/3.18	–	Delayed 2 d (owner scheduling)
wk 7	27 Jan 2025	30 Jan 2025	Cyclophosphamide 250 mg/m^2^ IV; prednisolone 0.5 mg/kg PO q24h × 7 d	27 Jan: 1.89/0.83; 30 Jan: 5.38/3.90	3	Withheld; filgrastim 27 Jan; resumed 30 Jan (3 d)
wk 8	6 Feb 2025	13 Feb 2025	Vincristine 0.6 mg/m^2^ IV (reduced); prednisolone 0.5 mg/kg PO q24h × 7 d	6 Feb: 3.48/1.98; 13 Feb: 5.41/3.00	1	Postponed 7 d (prior grade 3 event and owner scheduling); vincristine reduced
wk 9	20 Feb 2025	22 Feb 2025	Doxorubicin 1 mg/kg IV; prednisolone 0.5 mg/kg PO q24h × 7 d	20 Feb: 2.00/0.86; 22 Feb: 6.66/5.55	3	Withheld; filgrastim 20 Feb; resumed 22 Feb (2 d)

a Laboratory reference intervals: white blood cells (WBC), 2.87–17.02 × 10^9^/L; neutrophils, 2.30–10.29 × 10^9^/L. Where two dates are shown, the first is the count on which treatment was withheld and the second the count on which it was resumed. b Graded according to VCOG-CTCAE v2 [20]; the nadir neutrophil count was 0.83 × 10^9^/L. d: days Week 1 was defined as the date of the first administration (13 December 2024).

**Table 2:** Comparison of the present case with previously reported cats with tracheal lymphoma or other tracheal tumors managed surgically or medically, using the information reported in each publication.

Report	Signalment	Respiratory Status at Presentation (As Reported)	Tumor Type; location; Obstruction	Staging Reported	Intervention and Airway Management	Chemotherapy	Follow-up and Outcome
Present case	10-y SF Korean Shorthair, 4.0 kg	Lateral recumbency, open-mouth breathing, stridor; RR 48/min	B-cell lymphoma; cervical trachea (C3), dorsal membranous wall; minimum lumen 1.55 mm; ≈87% obstruction by CSA on non-contrast CT	Non-contrast whole-body CT; FeLV/FIV negative (postoperative); no abdominal US	R&A, 4 rings (≈16 mm); cross-field ventilation via temporary tracheotomy; clear, narrow margins	CHOP-based, 2 cycles (≈10 wk)	6.5 mo; no recurrence on contrast CT within field examined
Bataller et al. [12]	8-y SF Siamese	Bright and responsive; RR 50–60/min; mild inspiratory dyspnea, stridor	B-cell lymphoma; trachea; lumen reduced to 4 mm on tracheoscopy	FeLV/FIV negative; abdominal US declined (financial)	R&A, 7 rings; incomplete margins	COP, 6 wk	20 mo; no recurrence on tracheoscopy
Dugas et al. [11]	Cat (NR)	NR	Intratracheal lymphosarcoma	NR	None (medical only)	Systemic chemotherapy	Radiographic tumor reduction/remission
Kanemoto et al. [9]	Cat (NR)	NR	Lymphoma; trachea	NR	R&A	NR	Died 7 d postoperatively (suspected anastomotic dehiscence)
Miller et al. [14]	10-y MC DSH	Increased respiratory effort; occasional open-mouth breathing	SCC; trachea; incomplete margins	Regional lymph node excised	R&A, 5 rings (≈3 cm); cross-field ventilation via surgical site	None pursued	Well at 120 d

SF, spayed female; MC, male castrated; DSH, domestic shorthair; RR, respiratory rate; CT, computed tomography; US, ultrasonography; FeLV, feline leukemia virus; FIV, feline immunodeficiency virus; R&A, resection and anastomosis; SCC, squamous cell carcinoma; COP, cyclophosphamide, vincristine, and prednisolone; NR, not reported.

## Data Availability

The original contributions presented in this study are included in the article. Further inquiries can be directed to the corresponding author.

## Abbreviations

The following abbreviations are used in this manuscript:

CT	Computed tomography
G-CSF	Granulocyte colony-stimulating factor
CHOP	Cyclophosphamide, doxorubicin, vincristine, and prednisolone
EtCO_2_	End-tidal carbon dioxide
FeLV	Feline leukemia virus
FIV	Feline immunodeficiency virus
fSAA	Feline serum amyloid A
R&A	Resection and anastomosis
RI	Reference interval
VCOG-CTCAE	Veterinary Cooperative Oncology Group common terminology criteria for adverse events
